# Exploring online and in-person mental healthcare access and app use in a cohort of people living with disability: results from the 2019 and 2020 California Health Interview Survey

**DOI:** 10.1016/j.invent.2026.100931

**Published:** 2026-03-07

**Authors:** William Bevens, Jeongmi Kim, Biblia Cha, Nicole A. Stadnick, Elizabeth Eikey, Margaret Schneider, Stephen M. Schueller, Dana B. Mukamel, Dara H. Sorkin

**Affiliations:** a Department of Psychiatry, University of California San Diego, CA, USA; b Institute for Clinical and Translational Science, University of California Irvine, CA, USA; c Department of Psychological Science, School of Social Ecology, University of California, Irvine, USA; d University of California San Diego, Altman Clinical and Translational Research Institute Dissemination and Implementation Science Center, La Jolla, CA, USA; e Child and Adolescent Services Research Center, University of California San Diego, CA, USA; f Herbert Wertheim School of Public Health and Human Longevity Science, University of California, San Diego, San Diego, CA, USA; g The Design Lab, University of California, San Diego, San Diego, CA, USA; h Department of Public Health, University of California, Irvine, USA

**Keywords:** Disability, Mental health, mHealth, Digital health, Mental healthcare

## Abstract

**Background:**

Disability is an increasingly prevalent issue in the United States, which affects over 67 million people. Poor mental health in individuals with disabilities is common; however, access to traditional modes of mental healthcare remains a challenge.

**Objectives:**

This study aims to compare use of traditional and online mental healthcare services between people with and without disabilities.

**Methods:**

This study used a cross-sectional sample of adults aged 18 years and older (*n* = 44,096) from the 2019–2020 California Health Interview Survey. Mental healthcare access in-person and online, or use of digital technologies for mental healthcare were compared between several disability groups to individuals without disabilities.

**Results:**

15.3% of those in this sample reported being in one of the five reported disability groups: cognition, independent-living, seeing/hearing, self-care or multiple. Individuals with disabilities were 2.80 times more likely to access mental healthcare in-person compared to those without disabilities. Several disability groups had increased odds of accessing mental healthcare online, using online technologies for referrals to mental health professionals and connecting to others with a similar condition online.

**Conclusions:**

People with disabilities readily access mental healthcare, in-person and online, and use technologies for broader mental healthcare needs. This study highlights the importance of centering accessibility within health technologies.

## Highlights

• Individuals with disability readily access mental healthcare, in-person and online.
• Differences in accessing mental healthcare between sociodemographics were similar for those with and without disabilities.
• Technologies are used to support mental healthcare through treatment as well as referrals and peer support.

## 1 Introduction

Disability is a critical issue for the United States and was estimated to affect over 67 million people nationwide in 2019 (Varadaraj et al., 2021). Those living with disabilities experience higher rates of poor mental health outcomes, including a greater risk of psychological distress (Lundeen et al., 2022) and depression (Lewis et al., 2017). People living with disabilities, particularly younger and middle-aged adults, also access general and mental healthcare at lower rates compared with other groups (Okoro et al., 2018). Lack of healthcare access, including mental healthcare, is a major concern among adults living with disabilities (Okoro et al., 2018) and is associated with higher psychological distress and poorer social outcomes (Tough et al., 2017). Data have consistently demonstrated unmet mental healthcare needs among people living with disabilities (McColl et al., 2010), where traditional mental healthcare services are not available or fail to meet the needs of people with disabilities (Chaplin et al., 2009).

Barriers to accessing traditional modes of mental healthcare such as cost, physical access, difficulty navigating bureaucracy and difficulty scheduling appointments may be significant drivers of reported underutilization within this population (Trollor, 2014). In this context, “brick-and-click” approaches that blend traditional services with digital technology may facilitate greater access to mental healthcare for people with disabilities (Schueller, 2021). During the COVID-19 pandemic, it is estimated that between 39 and 45% of people with either a visual, auditory, mobility or cognitive disability used a telehealth service in the United States to access general, mental or other healthcare services (Xie et al., 2023a). Importantly, people with disabilities articulate the potential for digital mental healthcare to positively impact their lives (MacHale et al., 2023); however, disparities in the accessibility of these technologies remain.

The disability digital divide persists where people with disabilities are estimated to be less likely to access digital health technologies compared to those without disabilities (Pettersson et al., 2023). This may be in part due to the lack of accessibility tailoring within digital technologies such as telehealth or apps, including a lack of integration with visual aids or poorly implemented audio-supports (Henni et al., 2022; Jones et al., 2018). Importantly, as countries further integrate digital technologies into their health systems, lack of accessibility threatens to deepen health inequities for people with disabilities (Kessel et al., 2022). To prevent the entrenchment of an unequal mental healthcare system, data on the use of digital mental healthcare tools and services is critical for governments and policy makers to adequately prepare for the future.

The purpose of this study is to report mental healthcare access prevalences for those with and without disabilities, and to compare frequency of access of mental healthcare services and digital tools to support mental health between individuals with and without self-reported disabilities. We leverage data from the California Health Interview Survey (CHIS), the nation's largest statewide health survey, which allows us to investigate the relationship between disability and the self-reported use of in-person mental health services and digital mental healthcare tools.

## 2 Materials and methods

### 2.1 Data collection

This study is an analysis of the 2019 and 2020 California Health Interview Survey (CHIS), the largest statewide health survey in the US that examines population health and healthcare access issues in California. CHIS is a cross-sectional, mixed-mode (web and telephone) survey that uses an address-based sampling frame to recruit study participants. For all sampled households, one randomly selected adult in each sampled household either completed an on-line survey or was interviewed by telephone (UCLA Center for Health Policy Research, 2021). Data from the CHIS 2019 and 2020 surveys were collected between September 2019 and November 2020, of which approximately 90% of included data were completed over the web with the remainder over the phone. Surveys are administered in six languages: English, Spanish, Chinese (Mandarin and Cantonese dialects), Vietnamese, Korean, and Tagalog. Missing data were imputed and weights were applied to the sample data to produce population estimates from CHIS data (UCLA Center for Health Policy Research, 2021; UCLA Center for Health Policy Research K, 2021).

### 2.2 Measures

#### 2.2.1 Primary exposure: disability

CHIS queries disability according to the minimum standard set of questions developed for the American Community Survey (Bureau, 2021), implemented within a number federal government surveys such as the National Health Information Survey and the Behavioral Risk Factor Surveillance System surveys. The primary independent variable for this study is disability, which was assessed using the six-question sequence (6QS). This questionnaire was developed during an interagency working group in 2006 and implemented within the American Community Survey (Bureau, 2021), which identifies people living with disabilities within the population. The U.S. Department of Health and Human Services, through the Affordable Care Act, now mandates that all national population health surveys include these identifiers of disability at a minimum. These questions are a reliable indicator of disability in populations and have demonstrated agreement with other survey measures to identify people living with disabilities (Weeks et al., 2021). Overall, these data capture a diverse set of disabilities, such as individuals living with temporary, chronic or permanent disabilities as a result of disease or aging (Ward et al., 2017).

A modified version of the 6QS was administered within the CHIS dataset for 2019/2020 which omitted the mobility response and combined vision and deaf responses into one category. Three questions query difficulties related to Activities of Daily Living and one question queries respondents as to whether they are blind or deaf or have a severe vision or hearing problem. Respondents were categorized by their response to the following questions (Table 1): Cognition: “Because of a physical, mental, or emotional condition, do you have serious difficulty concentrating, remembering, or making decisions?” Self-care: “Do you have difficulty dressing or bathing?” Independent living: “Because of a physical, mental, or emotional condition, do you have difficulty doing errands alone such as visiting a doctor's office or shopping?” Vision/hearing: “Are you blind or deaf, or do you have severe vision or hearing problem?” A researcher derived category of ‘multiple disabilities’ was then derived, which contained responses from those who selected more than one disability category. Each disability group is coded mutually exclusive of each other for analysis.

**Table 1:** Number and percentages of included sample by sociodemographics.

Variable	N = 44,096	*Unweighted %*	*Weighted %*
Disability			
None	37,353	84.7	83.6
Cognition only	1907	4.3	5.4
Independent-living only	719	1.6	1.7
Seeing/hearing only	1992	4.6	3.9
Self-care only	234	0.5	0.4
Multiple	1,891	4.3	5.0
Age			
18–44 years	12,109	27.5	47.7
45–64 years	15,810	35.8	31.7
65+ years	16,177	36.7	20.6
Gender			
Female	24,748	56.1	50.9
Male	19,348	43.9	49.1
Race			
Black/African-American only, NH	1581	3.6	5.5
Asian only, NH	5292	12.0	13.3
Hispanic	8358	18.9	39.2
Other/two or more races, NH	1320	3.0	3.5
White, NH	27,545	62.5	38.5
Income			
Low	16,077	36.5	43.6
Medium	13,807	31.3	29.0
High	14,212	32.2	27.4

Legend: N = number.

#### 2.2.2 Dependent variables: mental healthcare access and technology use

Two questions were combined to assess access to in-person mental healthcare. The first question asked respondents if they had seen their primary care physician or general practitioner for problems for mental health, emotions, nerves, or their use of alcohol or drugs in the past 12 months. The second question asked if respondents had seen any other professional, such as a counselor, psychiatrist, or social worker over the same period for the same concerns. Respondents who answered ‘yes’ (1) to either or both questions were considered to have accessed healthcare for mental health or substance use concerns in the previous 12 months; else they were coded as ‘no’ (0).

Outcome variables for the three different remaining logistic regression models are as follows, and the response categories to each question is 1 (yes) or 0 (no). Variables were calculated for models according to the following questions respectively: “In the past 12 months, have you tried to get help from an online tool, including mobile apps or texting services for problems with your mental health, emotions, nerves, or your use of alcohol or drugs?” “In the past 12 months, have you connected online with people that have mental health or alcohol/drug concerns similar to yours through methods such as social media, blogs, and online forums?” “In the past 12-months, have you used online tools to find, be referred to, contact, or connect with a mental health professional?”

#### 2.2.3 Covariates

Covariates were guided by a priori knowledge of predictors of healthcare access and utilization (Recchia et al., 2022). As such, the following common sociodemographic and health outcome variables were included in all models: gender (male or female), age (18–44; 45–64; ≥65), ethnicity (white, non-Hispanic (NH); Hispanic; Asian only, NH; African-American only, NH) and annual household income (low ≤ $60,000; medium ≤ $120,000; high = ≥$120,000).

#### 2.2.4 Prevalence table variables

The outcome variable for the prevalence table calculations was mental healthcare access in the previous 12 months. This was assessed across two questions:

1. In the past 12 months have you seen your primary care physician or general practitioner for problems with your mental health, emotions, nerves, or your use of alcohol or drugs?
2. In the past 12 months have you seen any other professional, such as a counselor, psychiatrist, or social worker for problems with your mental health, emotions, nerves, or your use of alcohol or drugs?

If participants responded ‘yes’ to either or both questions, they were considered to have accessed healthcare for mental health or substances use concerns in the previous 12 months.

### 2.3 Statistical analysis

#### 2.3.1 Weighting

CHIS 2019/2020 data were weighted according to the process extensively outlined previously (UCLA Center for Health Policy Research, 2021). Briefly, the weighting procedure broadly aimed to compensate for differential probabilities of selecting for households and individuals within households, and to adjust for non-response and under-coverage within the sample.

#### 2.3.2 Data analysis

UCLA CHIS Data Access Center (DAC) performed analyses using SAS 9.4. The UCLA South General IRB has approved DAC to conduct analyses on confidential CHIS data (IRB#11-002227). It was considered possible that the emergence of COVID during 2020 may have affected mental health service and technology use between 2019 and 2020 and therefore, sensitivity analysis was used to explore differences in demographics and outcome measures between cohort 2019 and 2020. This analysis determined that these two cohorts did not differ across these variables and these datasets were appropriate to combine. Table 1 frequencies and percentages for demographics are presented; unweighted and weighted percentages are presented to demonstrate the impact of the weighted adjustments on distribution of demographics in the sample. Unadjusted prevalences and prevalence ratios (PR) were calculated for those who accessed an online tool in the past 12 months between those with and without disabilities across a range of sociodemographic variables. Data were stratified by disability vs no disability, and contingency tables were constructed between sociodemographic variables and access to mental healthcare within these strata. Odds ratios were estimated using multiple multivariable logistic regression models and odds ratios and 95% confidence intervals (CI) were calculated and presented for each coefficient. Whether to include interaction terms was guided by theory and assessed within models with F-test for significance, whereby no interaction terms were deemed appropriate to include in our models.

## 3 Results

### 3.1 Participant characteristics

In total, 44,096 adults surveyed in the 2019–2020 CHIS were included in this analysis. Overall, 15.3% of respondents reported being in one of the five disability groups-only (Table 1). Cognition was the most frequently reported disability category while self-care was the least frequently reported. A majority of the sample was female (50.9%) compared to male (49.1%).

### 3.2 Prevalence of in-person healthcare seeking for mental health or substance use concerns by disability status

Of those with disabilities 33.15% reported seeking mental healthcare from a GP, psychologist or other HCP in the previous 12 months compared with 11.82% of those without a disability (PR = 2.80) (Table 2). Females without disabilities were 51% more likely to access mental health services than males. For those with disabilities, females were 20% more likely to access mental health services compared with males. Differences between age groups for those with and without disabilities were similar with the ≥65 age group least likely to access mental health services compared with younger age groups.

**Table 2:** Prevalence of health care seeking for mental health care or substance use concerns by disability status, and prevalence ratios by demographics.

Characteristic	Adults with a disability % (CI)	PR (95% CI)	Adults without a disability % (CI)	PR (95% CI)
Overall	**33.15 (32.34–34.84)**	**2.80 (2.62–2.98)**	**11.82 (11.38–12.29)**	
Gender				
Male	29.80 (27.28–32.32)	Reference	9.42 (8.82–10.03)	Reference
Female	35.94 (33.46–38.43)	1.20 (1.08–1.35)	14.19 (13.51–14.88)	1.51 (1.39–1.63)
Age group				
18–44	43.32 (40.09–46.55)	Reference	14.81 (13.03–14.59)	Reference
45–64	33.45 (30.36–36.54)	0.77 (0.68–0.87)	10.11 (9.35–10.87)	0.68 (0.62–0.75)
≥65	18.28 (16.20–20.36)	0.42 (0.37–0.48)	7.06 (6.40–7.72)	0.48 (0.43–0.53)
Race/ethnicity				
White, NH	33.64 (31.56–35.72)	Reference	14.67 (14.04–15.32)	Reference
Hispanic	33.59 (30.33–36.86)	1.00 (0.89–1.12)	10.84 (9.90–11.78)	0.74 (0.67–0.81)
Black/African, NH	32.59 (24.54–40.64)	0.97 (0.75–1.25)	12.06 (9.66–14.46)	0.82 (0.67–1.01)
Asian, NH	28.50 (23.85–33.15)	0.85 (0.71–1.01)	6.14 (5.18–7.09)	0.42 (0.35–0.49)
Other/two or more races	34.87 (27.89–41.85)	1.04 (0.84–1.27)	14.34 (11.21–17.48)	0.98 (0.78–1.23)
Psychological distress				
Low	13.61 (11.43–15.78)	Reference	6.85 (6.40–7.31)	Reference
Moderate	39.29 (36.72–41.86)	2.89 (2.43–3.43)	20.81 (19.57–22.05)	3.04 (2.76–3.33)
Severe	56.34 (51.72–60.95)	4.14 (3.47–4.94)	32.89 (28.82–36.95)	4.80 (4.14–5.56)
Household income				
Low	31.64 (29.46–33.81)	Reference	10.66 (9.88–11.45)	Reference
Middle	34.70 (31.25–38.15)	1.10 (0.96–1.25)	11.35 (10.51–12.20)	1.07 (0.96–1.18)
High	37.32 (33.40–41.23)	1.18 (1.05–1.32)	13.84 (12.91–14.76)	1.30 (1.17–1.44)

Table 2. Prevalence of in-person health care seeking for mental health care or substance use concerns by disability status, and prevalence ratios by demographics.

Those reporting within the other/two or more races category had the highest prevalence of mental healthcare services access for those with a disability (prevalence = 34.87) and without disability (prevalence = 13.34). For those with disabilities, Hispanic respondents accessed mental health services at a similar rate to White, non-Hispanic respondents (PR = 1.00). Conversely, those reported as Hispanic without disabilities reported lower prevalence of accessing mental healthcare (PR = 0.74). Those with disabilities in the Asian, non-Hispanic category had a lower prevalence to those reporting white, non-Hispanic (PR = 0.85). Comparing these two groups for those without disabilities, the Asian, non-Hispanic group described a lower prevalence compared to White, non-Hispanic respondents (PR = 0.42). For those reporting severe psychological distress, the prevalence for those with disabilities and those without disabilities was 56.34 and 32.89 respectively.

### 3.3 Associations between disability and accessing mental healthcare services or using technology to support mental health

Table 3 describes the adjusted odds ratios of accessing mental healthcare services or using technology to support mental health across four regression models. Model 1 reported that all disability groups except for self-care were associated with greater odds of seeking mental healthcare in-person compared to those who did not report disability after adjustment: those with multiple or a cognition disability were almost six times more likely to seek mental healthcare in-person compared to those without disability (OR 5.92; CI 5.00; 7.01 & OR 5.78; CI 4.93; 6.77, respectively). Respondents who reported an independent living disability were over three times more likely (OR 3.53; CI 2.79; 4.47) while those with a seeing/hearing disability were 1.4 times more likely (OR 1.41; CI 1.09; 1.82) to seek mental healthcare in-person compared to those without disabilities. For model 2, those who reported a cognition disability described over three-and-a-half times greater odds of seeking mental healthcare online (OR 3.88; CI 3.17; 4.75), multiple disabilities was associated with an over two-and-a-half greater odds (OR 2.74; CI 2.12; 3.53) while independent-living disability was associated with just under two-and-a-half times greater odds (OR 2.41; CI 1.74; 3.34).

**Table 3:** Associations between disability and accessing mental healthcare services/using technology to support mental health.

	Model 1: Seek mental healthcare in-person	Model 2: Seek mental healthcare online	Model 3: Connect with others similar online	Model 4: Referred to professionals online
	***Adjusted OR (95% LCI; UCI)***			
***Primary exposure***				
**Disability:** None	Reference	Reference	Reference	Reference
Cognition	5.78 (4.93; 6.77)⁎	3.88 (3.17; 4.75)⁎	4.40 (3.54; 5.47)⁎	3.43 (2.85; 4.13)⁎
Independent-living	3.53 (2.79; 4.47)⁎	2.41 (1.74; 3.34)⁎	3.13 (2.15; 4.56)⁎	3.17 (2.32; 4.32)⁎
Seeing/hearing	1.41 (1.09; 1.82)⁎	0.78 (0.53; 1.16)	0.85 (0.37; 1.97)	1.33 (0.97; 1.82)
Self-care	1.50 (0.87; 2.57)	1.63 (0.76; 3.47)	0.86 (0.30; 2.47)	1.21 (0.47; 3.14)
Multiple	5.92 (5.00; 7.01)⁎	2.74 (2.12; 3.53)⁎	4.00 (3.05; 5.25)⁎	2.42 (1.93; 3.05)⁎
***Sociodemographic confounders***				
**Race:** White, NH	Reference	Reference	Reference	Reference
African only, NH	0.78 (0.62; 0.98)⁎	0.95 (0.72; 1.26)	0.85 (0.56; 1.29)	0.93 (0.71; 1.22)
Asian only, NH	0.39 (0.34; 0.45)⁎	0.84 (0.70; 1.00)	0.58 (0.46; 0.72)⁎	0.54 (0.43; 0.67)⁎
Hispanic	0.65 (0.59; 0.72)⁎	0.83 (0.71; 0.98)⁎	0.65 (0.55; 0.77)⁎	0.75 (0.64; 0.87)⁎
Other/≥2 races, NH	0.80 (0.65; 0.99)⁎	1.05 (0.74; 1.49)	0.84 (0.60; 1.17)	0.98 (0.74; 1.29)
**Age:** 18–44 years	Reference	Reference	Reference	Reference
45–64 years	0.63 (0.58; 0.70)⁎	0.45 (0.39; 0.52)⁎	0.32 (0.26; 0.39)⁎	0.43 (0.37; 0.49)⁎
65+ years	0.35 (0.31; 0.39)⁎	0.13 (0.10; 0.15)⁎	0.10 (0.07; 0.14)⁎	0.15 (0.12; 0.19)⁎
**Gender:** Male	Reference	Reference	Reference	Reference
Female	1.60 (1.48; 1.73)⁎	1.42 (1.25; 1.61)⁎	1.65 (1.40; 1.94)⁎	1.50 (1.32; 1.70)⁎
**Annual household income:** Low	Reference	Reference	Reference	Reference
Middle	1.07 (0.96; 1.19)	1.03 (0.87; 1.22)	0.78 (0.65; 0.93)⁎	1.25 (1.05; 1.48)⁎
High	1.28 (1.14; 1.42)⁎	1.03 (0.87; 1.23)	0.64 (0.52; 0.79)⁎	1.59 (1.37; 1.86)⁎


*Model fit statistics*	*F*	Df	*p*	*F*	Df	*p*	*F*	Df	*p*	*F*	Df	*p*
Likelihood ratio	368.5	12.6	<0.001	171.5	12.3	<0.001	163.8	11.4	<0.001	156.6	11.6	<0.001
Lagrange multiplier	96.0	14	<0.001	43.35	14	<0.001	30.2	14	<0.001	46.8	14	<0.001
Wald	114.6	14	<0.001	52.43	14	<0.001	50.0	14	<0.001	44.8	14	<0.001

Legend: F = F-statistic; Df = degrees of freedom; OR = odds ratio; LCI = lower bound confidence interval; UCI = upper bound confidence interval; *p* *= p*-value.

⁎ Statistically significant (*p* < 0.05).

For model 3, connecting online with others who had a similar mental health concern was associated with almost four-and-a-half times greater odds for those who reported a cognition disability (OR 4.40; CI 3.54; 5.47), more than three times greater odds for those who reported an independent-living disability (OR 3.13; CI 2.15; 4.56), and four times greater odds for those who reported multiple disabilities (OR 4.00; CI 3.05; 5.25). For model 4, those who reported a cognition or independent-living disability had over three times greater odds of using the internet to be referred to professionals compared to those without disabilities (OR 3.17; CI 2.69; 3.73 & OR 3.17; CI 2.32; 4.32, respectively). Those who reported multiple disabilities described under two-and-a-half times greater odds (OR 2.42; CI 1.93; 3.05).

## 4 Discussion

This study aimed to report on mental healthcare access prevalences for those with and without disabilities, and to compare the use of mental healthcare services and digital tools to for mental healthcare between those who self-reported disabilities and those who did not. Firstly, we report recent data on the rates at which individuals with and without disability access mental healthcare services using a large, representative dataset. Secondly, we describe novel data, which estimates that individuals with disabilities are more likely to use a range of in-person and digital services to support their mental health compared to those without disabilities. This previously unreported data is critical to support researchers and policy makers in the development of digital infrastructure to support the mental healthcare of people with disabilities. Importantly, we provide disability-group specific data, which is necessary to facilitate tailoring of these services for different populations.

### 4.1 Comparison with prior work

In comparison to persons who did not report a disability, this study showed higher in-person mental healthcare-seeking among persons with a range of disabilities, and higher online mental healthcare seeking for those with a cognition, independent living or multiple disabilities. In this context, these data suggest a strong demand for mental health services among people with a range of disabilities. Individuals with disabilities already face difficulties maintaining health insurance and forego healthcare (Xie et al., 2023b), and this highlights the potential for a mismatch between demand and access. There is scant, recent data on the frequency at which people who report disabilities access in-person mental healthcare services compared to those without disabilities.

A 2022 study using National Survey on Drug Use and Health data from 2015 to 2019 reported a higher prevalence of mental health services use for those with disabilities compared to those without in a sample of people who reported a Serious Mental Illness (SMI) in the prior year (Xie et al., 2022). Conversely, a Canadian study reported that those with intellectual disabilities ins Québec accessed psychological services at similar rates to those without disabilities (Maltais et al., 2020); however, this study had a smaller sample than the National Survey on Drug Use and Health. Other studies with relevant data have focused on frequencies of general health service access as opposed to mental health service access for people with disabilities with and without mental health disorders (Okoro et al., 2018); combined populations of people with disabilities with other non-disabling chronic conditions to assess mental health service access (Andrews et al., 2001); or delayed or forgone mental healthcare (Xie et al., 2023b). It is possible that disability groups of seeing/hearing were comparatively more likely to seek in-person compared with online mental healthcare due to accessibility limitations within the technologies themselves (Khan and Khusro, 2021). Another possible mechanism for increased mental healthcare seeking behavior among persons with disabilities would be that these individuals have more frequent engagement with the broader healthcare system in general (Maltais et al., 2020). This increased interaction with the healthcare system could lead those with disabilities to develop skills in self-efficacy and self-management earlier than those without disabilities leading to increased healthcare system engagement (Thompson et al., 2012). This may be especially true for those who reported multiple disabilities where they would be the group most likely to engage multiple medical providers to address their complex health needs. Further, it is possible that psychological distress mediates the relationship between disability and mental healthcare seeking behavior (Cree et al., 2020). Subgroup analyses of people with disabilities with low psychological distress may yield comparable odds of engaging with in-person and online mental healthcare as to those without disabilities.

All disability groups except for seeing/hearing and self-care were much more likely to use online resources to engage with those who share a similar mental health condition. People with disabilities have smaller social networks and experience social isolation and loneliness at higher rates compared to those without disabilities (Emerson et al., 2021). As such, prior data suggests that people with disabilities use the internet and social media frequently (Glencross et al., 2021), sometimes more frequently than those without disabilities (Dobransky and Hargittai, 2021). Our findings are consistent with these results for all disability groups excluding seeing/hearing and self-care, who reported similar rates of engaging with peers online compared to those without disabilities. Particularly for those in the multiple disability groups the internet may provide an important tool to connect with less common permutations of symptoms and functional limitations. The differences in the seeing/hearing disability group may be a result of differential use of technology. While many of those who are blind/visually impaired or deaf/hard of hearing engage with the internet and social media and report positive outcomes from doing so (Fuglerud et al., 2012), audio and visual content has increasingly replaced text-based content across social media websites and apps, which often negatively affects this disability group specifically (Voykinska et al., 2016). For people with vision impairment or blindness, connecting with others over shared mental health conditions is highly dependent on environmental factors (van Munster et al., 2021) and therefore, a lack of difference here may indicate inadequate platform accessibility. Importantly, platforms are increasingly employing accessibility features within video content that may continue to address challenges raised by individuals represented in the seeing/hearing disability group (Voykinska et al., 2016). This is critically important for individuals in with seeing/hearing disabilities who report unmet needs when it comes to mental healthcare (Xie et al., 2023c).

Much of the existing data on how people with disabilities engage with referral systems for mental healthcare has focused on adults with intellectual disabilities (Whittle et al., 2018). This study demonstrates that compared to those without disability, individuals with a cognition, independent living or multiple disabilities are more likely to use online tools to find, be referred to, contact, or connect with a mental health professional. These results are consistent with the increased mental healthcare seeking behavior described for people with disabilities earlier. The seeing/hearing and self-care disability groups reported no difference to those without disabilities for use of online tools to find, be referred to, contact, or connect with a mental health professional, similar for online mental healthcare seeking in this group. It is possible that because the self-care disability group consists of many people with physical disabilities (Hauenstein et al., 2022), these individuals seek out community support in preference to support from professionals. For those with seeing/hearing disabilities, they may preference in-person referrals where navigating online forums is challenging and less trusted (Voykinska et al., 2016).

In an increasingly digitalized healthcare system, better understanding of the needs of people with disabilities to navigate online mental health services is a priority. The timing of these data suggests that these may not be merely pandemic-specific effects but a pattern that existed prior. Other studies have described a ‘digital divide’ for people with disabilities where accessing online health information and patient portals is considered more difficult compared to those without disabilities (Pettersson et al., 2023). These data may suggest a tension between people with disabilities increasingly sourcing online health information and care while also experiencing disproportionate difficulty engaging these resources. Efforts are already underway within the US and abroad to ensure that the digital future of healthcare is centered on people living with disabilities so as to appropriately incorporate their needs (Kessel et al., 2022). This has been less explored within the context of mental healthcare and remains a crucial next step.

### 4.2 Limitations

Specific to this dataset, one question regarding the functional domain ‘mobility’ was not collected during this wave. Therefore, this dataset underestimates the prevalence of disability within this population, particularly where a disability drives functional limitations in mobility (i.e., climbing stairs, walking on carpet). Interpretation of results should take into consideration that mobility was the largest reported functional domain in previous surveys (Cree et al., 2020). Additionally, seeing and hearing functional domains are combined for this dataset and therefore, individual associations and prevalence data are not able to be investigated. Further, this is not a clinically validated measure for ADLs, which may limit the generalizability of the outcomes from this study. Despite this, this is a frequently used measure across large local, state, and national surveys and is recognized as a useful indicator of disability. Many existing, validated scales are available that query between 6 and 29 items related to basic and complex tasks (Pashmdarfard and Azad, 2020). Another limitation is the dichotomization of disability groups. It is possible that as disabilities accumulate, this impacts patterns of use of mental healthcare and digital tools to support mental health. We chose to dichotomize disability into ‘yes or no’ across all disability groups as opposed to including every permutation of the combinations of disabilities reported. The latter would require too small samples for crosstabs and therefore, the former was adopted.

### 4.3 Conclusions

This study provides novel data on mental healthcare seeking for people across disability groups. Contrary to previous data, we demonstrate that all disability groups readily access mental healthcare, whether in-person or online. Importantly, we demonstrate that individuals with disabilities are more likely to access these services compared to those who do not report disabilities. These data will support clinicians and policy makers to guide mental healthcare into its digital future.

## CRediT authorship contribution statement

WB: Conceptualization, Data analysis, Methodology, Visualization, Writing and Editing – original draft; JK: Methodology, Data exploration and analysis, Writing and Editing; BC: Writing and Editing; NAS: Conceptualization, Writing and Editing; EE: Writing and Editing; MS: Writing and Editing; SMS: Methodology, Data analysis Writing and Editing; DBM: Writing and Editing; DHS: Conceptualization, Methodology, Data analysis Writing and Editing.

## Institutional review board statement

The UCLA South General IRB has approved DAC to conduct analyses on confidential CHIS data (IRB#11-002227).

## Declaration of Generative AI and AI-assisted technologies in the writing process

No Generative AI or AI-assisted technologies were used in the writing process for this manuscript.

## Funding

This work was funded by the 10.13039/100006108National Center for Advancing Translational Sciences of the National Institutes of Health (UL1TR001414).

## Declaration of competing interest

SMS has received consulting payments from Otsuka Pharmaceuticals and Boehringer Ingelheim and is a member of the Headspace Scientific Advisory Board, for which he receives compensation. No other authors declare a conflict of interest.

## Data Availability

Analysis code is available upon reasonable request to corresponding author. Certain data analyzed in this study is subject to restrictions in access as per California Health Interview Survey and UCLA regulations due to potential identifiability and confidentiality.
